# A Cotton (*Gossypium hirsutum*) *Myo*-Inositol-1-Phosphate Synthase (*GhMIPS1D*) Gene Promotes Root Cell Elongation in *Arabidopsis*

**DOI:** 10.3390/ijms20051224

**Published:** 2019-03-11

**Authors:** Rendi Ma, Wangyang Song, Fei Wang, Aiping Cao, Shuangquan Xie, Xifeng Chen, Xiang Jin, Hongbin Li

**Affiliations:** 1 College of Life Sciences, Key Laboratory of Xinjiang Phytomedicine Resource and Utilization of Ministry of Education, Shihezi University, Shihezi 832003, China; mrendi118@163.com (R.M.); swywinner@163.com (W.S.); wangfshzu@163.com (F.W.); aiping9smile@sina.com (A.C.); xiesq@shzu.edu.cn (S.X.); cxf_cc@shzu.edu.cn (X.C.); 2 Ministry of Education Key Laboratory for Ecology of Tropical Islands, College of Life Sciences, Hainan Normal University, Haikou 571158, China

**Keywords:** MIPS, exon-intron structure diversity, *Gossypium hirsutum*, loss-of-function mutant, root cell elongation

## Abstract

*Myo*-inositol-1-phosphate synthase (MIPS, EC 5.5.1.4) plays important roles in plant growth and development, stress responses, and cellular signal transduction. *MIPS* genes were found preferably expressed during fiber cell initiation and early fast elongation in upland cotton (*Gossypium hirsutum*), however, current understanding of the function and regulatory mechanism of *MIPS* genes to involve in cotton fiber cell growth is limited. Here, by genome-wide analysis, we identified four *GhMIPS* genes anchoring onto four chromosomes in *G. hirsutum* and analyzed their phylogenetic relationship, evolutionary dynamics, gene structure and motif distribution, which indicates that *MIPS* genes are highly conserved from prokaryotes to green plants, with further exon-intron structure analysis showing more diverse in *Brassicales* plants. Of the four *GhMIPS* members, based on the significant accumulated expression of *GhMIPS1D* at the early stage of fiber fast elongating development, thereby, the *GhMIPS1D* was selected to investigate the function of participating in plant development and cell growth, with ectopic expression in the loss-of-function *Arabidopsis mips1* mutants. The results showed that *GhMIPS1D* is a functional gene to fully compensate the abnormal phenotypes of the deformed cotyledon, dwarfed plants, increased inflorescence branches, and reduced primary root lengths in *Arabidopsis mips1* mutants. Furthermore, shortened root cells were recovered and normal root cells were significantly promoted by ectopic expression of *GhMIPS1D* in *Arabidopsis mips1* mutant and wild-type plants respectively. These results serve as a foundation for understanding the *MIPS* family genes in cotton, and suggest that *GhMIPS1D* may function as a positive regulator for plant cell elongation.

## 1. Introduction

*Myo*-inositol-1-phosphate synthase (MIPS; EC 5.5.1.4) is the rate-limiting enzyme to control *myo*-inositol (Ins) biosynthesis by converting d-glucose 6-phosphate (G6P) to inositol phosphate, followed by dephosphorylation reaction that is catalyzed by *myo*-inositol monophosphatase (IMP) [1]. The *MIPS* genes are widely spread in numerous organisms, from cyanobacteria to eubacteria and archaea, and ultimately to higher eukaryotes, such as higher plants and humans [2]. Numbers of *MIPS* have been identified in higher plants, including *Phaseolus vulgaris* [3], *Oryza sativa* [4], *Citrus paradise* [5], *Arabidopsis thaliana* [6], *Zea mays* [7], *Brassica napus* [8], *Glycine max* [9], and *Sesamum indicum* [10]. Protein sequence analysis of MIPS indicated that the eukaryotic MIPS family is homogenous, but different from the prokaryotic MIPS proteins [11]. This may be explained by the monophyletic origin of the eukaryotic *MIPS* genes. All eukaryotic MIPS sequences (around 510 amino acids) have shown regions of high conservation even at the nucleotide level [12], indicating that MIPS is very important for biological processes, such as embryogenesis and seed formation. There are four highly conserved amino acid sequences in MIPS proteins: GWGGNNG (domain 1), LWTANTERY (domain 2), NGSPQNTFVPGL (domain 3) and SYNHLGNNDG (domain 4) [1]. It is reported that these domains are involved in MIPS protein binding for catalyzing enzyme reaction and are essential for MIPS functions [2].

By catalyzing the biosynthesis of Ins and its derivates, including Ins polyphosphates (IPs), phospholipid phosphatidylinositol (PtdIns) and phosphoinositide phosphates (PtdInsPs), MIPS performs crucial diverse roles in biotic- and abiotic-stress responses, plant growth and organ development, and cell division [13]. The first MIPS was reported from *Archaeoglobus fulgidus* and might function under high temperatures [14]. In *Arabidopsis*, three genes encoding MIPS proteins (MIPS1–3) have been identified and appeared to have undergone functional divergence to some extent. Murphy et al. [15] found that loss-of-function *Arabidopsis mips2* mutants showed enhanced susceptibility to diverse viral, bacterial, and fungal pathogens, whereas *mips1* mutants showed no such effect. Furthermore, *mips1* mutants exhibit spontaneous cell death and enhanced resistance to the oomycete pathogen [16]. The *misp1* mutants were also reported to be sensitive to strong light stress [17]. The *mips1 mips2* double mutant and *mips1 mips2 mips3* triple mutant were embryo lethal, whereas the single *mips* mutants showed no obvious phenotype [18]. Severe reduction of MIPS activity inhibited plant growth significantly [19]. Lower IP_6_ generated by suppressing *MIPS* using RNA interference (RNAi)-mediated approach in potato led to the alteration of leaf morphology, induction of leaf senescence, and reduction of tuber yield [7].

Allotetraploid cotton *Gossypium hirsutum* is the most widely planted cotton species, which provide more than 90% of natural fiber for the textile industry. The yield of cotton fiber is greatly influenced by the fiber initiation and early stage of fiber elongation. Ins and its derivates also play important role in cell growth by involving in cell wall synthesis, membrane trafficking, and signal transduction. The oxidation product of Ins, d-glucuronic acid (GlcA), is used for cell wall pectic noncellulosic compounds and, in some organisms, for the synthesis of ascorbic acid (AsA) [20,21,22,23,24]. Pectin precursors were reported to be essential for cotton fiber elongation in tetraploid cotton *G. hirsutum* [25]. Our previous works presented that AsA and its oxidation metabolism catalyzed by ascorbate peroxidase (GhAPX) were important for cotton fiber development in *G. hirsutum* [26,27]. PtdIns and PtdInsP were significantly accumulated in fast elongating fibers and promoted fiber cell elongation in vitro [28]. *GhMIPS* genes were preferably expressed during fiber initiation and the early stage of fiber elongation development. Nonetheless, current understanding of cotton *MIPS* family genes and their functions involving in plant growth and development and fiber cell growth is limited. In this work, we identified four *MIPS* gene family members in *G. hirsutum* through a genome-wide investigation and characterized the detailed information of phylogenetic relationship, evolutionary dynamics, exon-intron structure, and conserved motif distribution. Furthermore, after consideration of the expression levels of the four *GhMIPS* genes during fiber development, we selected the *GhMIPS1D* to further investigate its functional roles in participating in plant development and cell growth by ectopic expression of *GhMIPS1D* in *Arabidopsis mips1* mutants. The results indicated that *GhMIPS1D* could rescue the mutant abnormal phenotypes and ectopic expression of *GhMIPS1D* in *mips1* mutant and wild-type (WT) *Arabidopsis* plants promoted the root cell elongation significantly.

## 2. Results

### 2.1. Characterization, Phylogenetic Relationship and Motif Distribution Analyses of GhMIPS Gene Family Members in G. hirsutum

Cotton *MIPS* family genes were characterized by submitting the *Arabidopsis* MIPS protein sequences against *G. hirsutum* genome (allotetroploid, AADD) database derived from Phytozome (v12.1.6), followed by conserved domain recognition using InterProScan. Four *GhMIPS* members were determined: *GhMIPS1A*, *GhMIPS2A*, *GhMIPS1D* and *GhMIPS2D* (the sub-genomes were indicated by A and D). The *GhMIPS1A* located in sub-genome A chromosome 2 while *GhMIPS1D* located in sub-genome D chromosome 3, indicating homologous recombination events might occur during the individual evolution of A and D sub-genomes (Supplementary Figure S1). Due to the highly conserved gene sequences and structures, all *GhMIPS* homologues were considered as duplicates and the duplication relationships were indicated by red lines in Supplementary Figure S1. The *Ka/Ks* analysis showed that all *GhMIPS* members are under purifying selection (Supplementary Table S1). Expression patterns of *GhMIPS* family genes showed that *GhMIPS1A* and *GhMIPS1D* were the most highly expressed members in cotton fibers, with the highest expression level at 0 day post anthesis (DPA) (no fiber growth) and 3 DPA (fiber initiation growth) (Supplementary Figure S2), implying that *GhMIPS1D* may perform potential important role in fiber growth and development.

**Figure 1:**
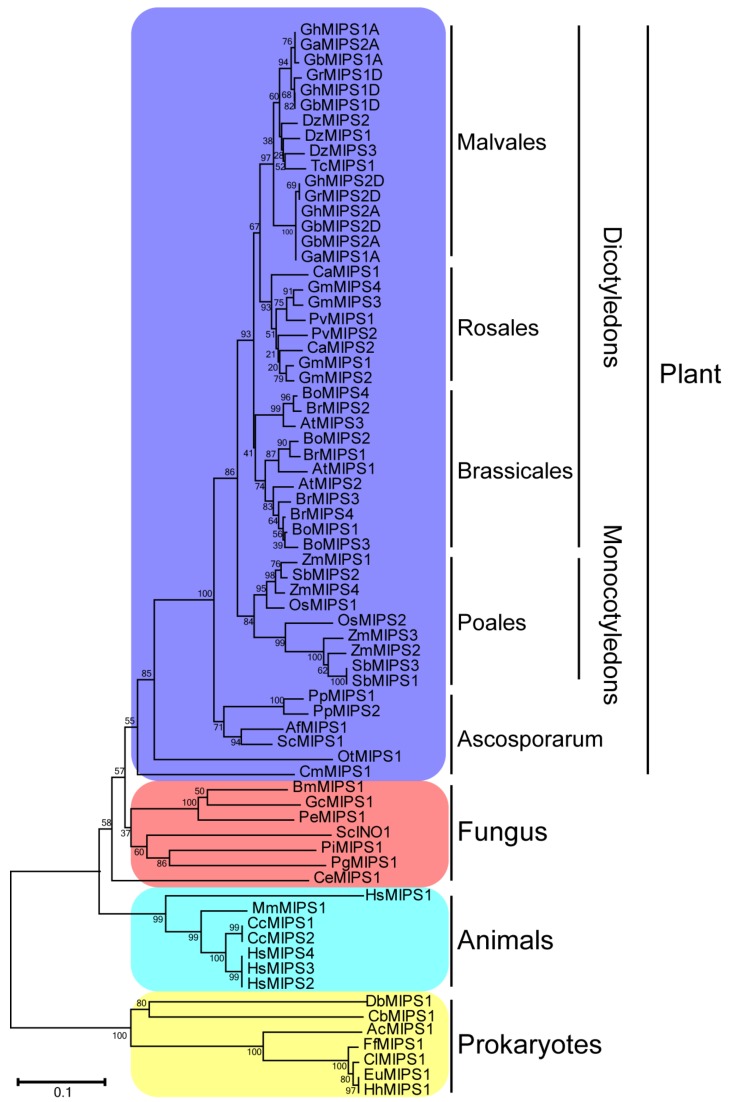
Phylogenetic tree of 70 MIPS homologues of 36 different species from prokaryotes to higher plants. See Supplementary Table S2 for detailed information of organism and protein sequences. The phylogenetic tree was constructed by MEGA5.0 using neighbor-joining method with bootstrap tests 1000.

Phylogenetic tree of 70 MIPS homologues of 36 different species from prokaryotes to higher plants was constructed to investigate the evolutionary characters of the *MIPS* gene family. In general, seven prokaryotes, seven animal, six fungus and fifty plant MIPS proteins were included for the phylogenic analysis. Detailed information of MIPS used in this study is provided (Supplementary Table S2), containing organism names, accession numbers and sequences. The *MIPS* gene family is a very small one, usually including one or two members in most plant species, except for *Z. mays* (diploid, monocotyledons); *Brassica rapa*, *Brassica oleracea* and *Glysine max* (diploid, dicotyledons); *G. hirsutum* and *Gossypium barbadense* (tetraploid, dicotyledons); which have four *MIPS* members each. In addition to *B. rapa* and *B. oleracea*, the model plant *A. thaliana* has three *MIPS* members, indicating that this protein family has expanded in most *Brassicales* plants (Figure 1).

**Figure 2:**
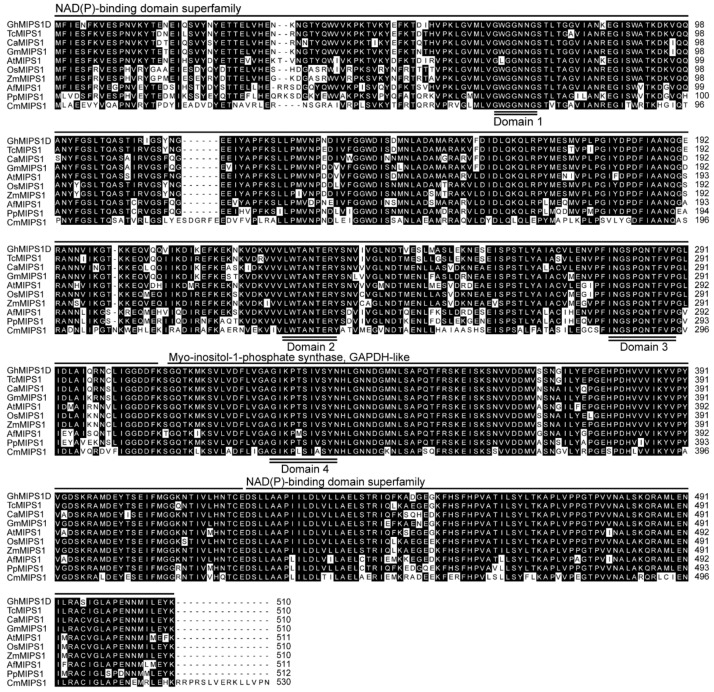
Multiple sequence alignment of ten representative MIPS proteins. See Supplementary Table S2 for organism and sequence information. Conserved domains are indicated with double underline (Domain 1–4).

**Figure 3:**
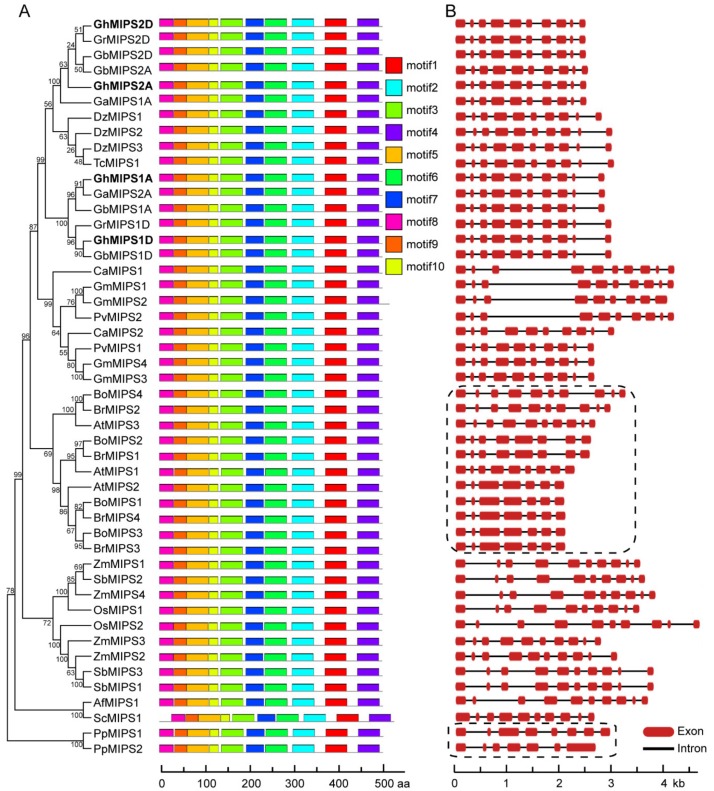
Conserved motif and exon-intron dynamic analyses of 48 plant *MIPS* genes. (**A**) Conserved motifs of protein sequences of 48 plant MIPS proteins. Ten conserved motifs were recognized and represented in different colors. All MIPS proteins are arranged according to their phylogenetic relationships. (**B**) The exon-intron structures of 48 plant *MIPS* genes. Two frames represent *Bryophytes* plants (*Physcomitrella patens*, seven and eight exons) and *Brassicales* plants (from seven to ten exons), which possess usual exon-intron structures. The detailed information for organism names, protein sequences and accession numbers are available in Supplementary Table S2.

GhMIPS1D and other nine typical plant MIPS proteins were used to perform multiple sequence alignment and conserved domain analysis, showing that MIPS proteins are quite conserved in plants with amino acid identity over 80%. Similar with the other nine plant MIPS proteins, cotton GhMIPS1D possessed two NAD(P)-binding domains distributing at the N and C terminus respectively, and one *myo*-inositol-1-phosphate synthase domain locating at the middle region, as well as four conserved domains that are responsible for MIPS protein binding and are essential for MIPS function exertion (Figure 2). Note that in higher plants, all MIPS proteins have around 510 amino acids, while CmMIPS1 of simple single cell algae (*Cyanidioschyzon merolae*) has 530 amino acids (Figure 2). Motif distribution analysis recognized ten conserved motifs that are organized similarly in all plant MIPS proteins (Figure 3A and see Supplementary Figure S3 for detailed sequences for motifs). These data suggest that MIPS is a highly conserved protein family in different plants, from ascosporarum to higher plants.

### 2.2. Exon-Intron Structure Evolution Analysis of MIPS Gene Family

To further study the evolutionary relationship of *MIPS* family members, exon-intron structure dynamic analysis of four cotton *GhMIPS* members and other 44 plant *MIPS* genes was performed. Only one exon was found in single-cell algae *C. merolae* and *Ostreococcus tauri*. However, *Physcomitrella patens PpMIPS1* and *PpMIPS2* possess eight and seven exons respectively, indicating that gene family expansion and gene structure variation occurred after the terrestrial plant has been evolved (Figure 3B). For higher plants, all MIPS proteins have ten exons except *B. rapa*, *B. oleracea* and *A. thaliana*, suggesting that *Brassicales* plants have undergone some special evolutionary events (Figure 3B, framed).

**Figure 4:**
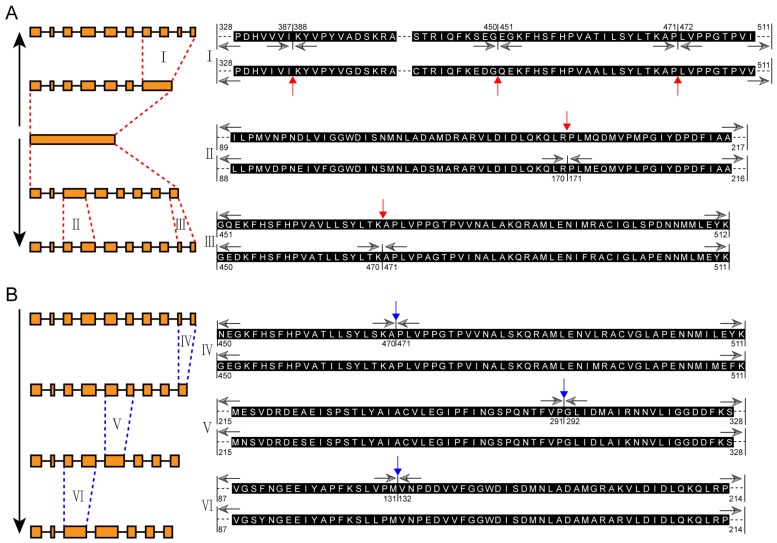
Illustration of the evolution model of *MIPS* exon-intron structures. (**A**) The evolution model of exon split events. *MIPS* possesses one entire exon in single cell algae. One evolution event has been recognized in *Bryophytes* plants, leading to a seven-exon *PpMIPS2* (up-arrow) and an eight-exon *PpMIPS1* (down-arrow). Then some of the exons have been split into more exons, forming the predominant exon-intron structure in higher plants (events I, II and III). The exon split sites are indicated by red arrows and amino acid numbers. (**B**) The evolution model of exon merge events in *Brassicales* plants. Three exon merge events have been identified, indicating there were three independent evolutionary events during the evolution of *Brassicales* plants. The exon-merge sites are indicated by blue arrows.

According to the phylogenetic tree and the exon-intron structures, an evolution model of the exon-intron structures for plant *MIPS* gene family was illustrated. The single-exon structure had been split into seven and eight exons shortly after the appearance of land plants. Then the last exon of the seven-exon structure homologues split into four new exons (event I, Figure 4A), while the 3rd and the last exons of the eight-exon structure members split into two new exons each (event II and event III, Figure 4A). Red arrows in Figure 4A indicate the exon split sites and black arrows indicate the amino acid sequences corresponding to the final exon-intron structures. Our data show that the formation of the ten-exon structure of *MIPS* genes is no later than arise of ferns (*Azolla filiculoides* and *Salvinia cucullata*).

Notably, three exons merge events were recognized in *Brassicales* plants, leading to the formation of nine-exon (*AtMIPS1*), eight-exon (*BoMIPS2* and *BrMIPS1*) and seven-exon (*AtMIPS2*, *BoMIPS1*, *BoMIPS3*, *BrMIPS3* and *BrMIPS4*) *MIPS* family members, respectively (Figure 4B, and Figure 3B). Interestingly, the three exons merge events seem to occur in order (from event IV to event VI), indicating the *Brassicales* plants *MIPS* might have undergone three special evolutionary events.

### 2.3. Functional Complementary Analysis of GhMIPS1D in the Loss-of-Function Arabidopsis mips1 Mutant

In the light of Ins and its derivates important functions in plant development and cell growth [13], as well as the accumulated expression of *GhMIPS1D* during cotton fiber development (Supplementary Figure S2), thus, the overexpression vector *35S::GhMIPS1D-GFP* generated by cloning the *GhMIPS1D* into the modified *pCAMBIA2300-GFP* vector, was transformed into the loss-of-function *mips1* mutant and WT *Arabidopsis* plants, to investigate the *GhMIPS1D* in vivo functions. The *35S::GhMIPS1D-GFP* vector was introduced into onion epidermal cells to determine the subcellular localization of GhMIPS1D. The results showed that GhMIPS1D located in the nucleus, plasma membrane and endomembranes (Supplementary Figure S4), suggesting its likely functions in membrane trafficking and signal transduction, and regulation of gene expression.

**Figure 5:**
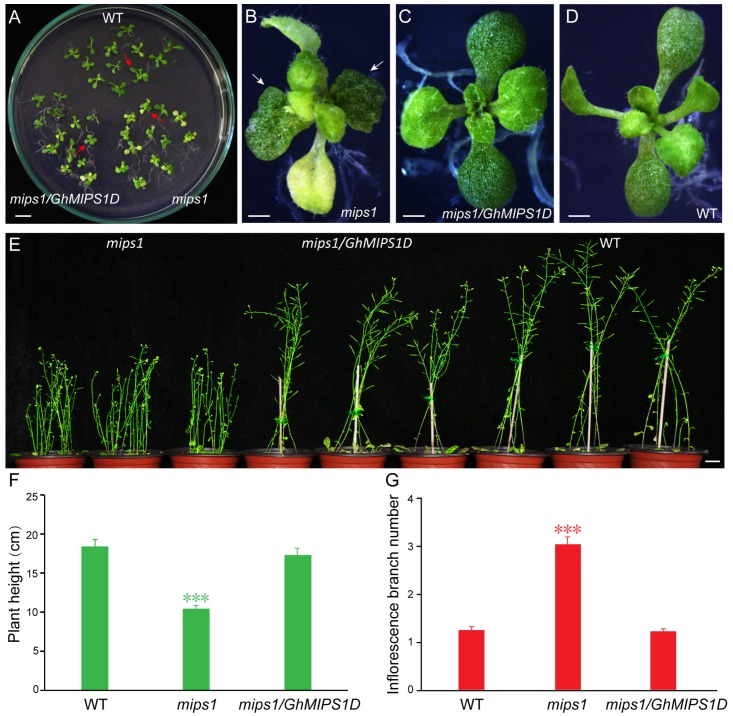
Ectopic expression of *GhMIPS1D* in *Arabidopsis* rescues the abnormal defects of *mips1* mutants. (**A**) The *mips1* mutant (*mips1*), transgenic (*mips1/GhMIPS1D*) and wild-type (WT) *Arabidopsis* plants grew on 0.5 × Murashige and Skoog (MS) medium for two weeks. Bar = 1 cm. (**B**–**D**), the enlarged image of indicated plants (red arrows in (**A**)). White arrows indicate the abnormal cotyledon in *mips1* mutants. Bars = 1 mm. (**E**) Phenotypes of *Arabidopsis* plants grew for ten weeks. Significant shorter plants and more inflorescence branches are observed in *mips1* mutants, but not in *mips1/GhMIPS1D* transgenic lines and WT plants. Bar = 1 cm. The plant height (**F**) and the number of inflorescence branches (**G**) are measured (*n* = 30). ***, *p* < 0.001 compared to WT.

The loss-of-function *Arabidopsis mips1* mutants were selected to perform genetic functional analysis of *GhMIPS1D*. The expression levels of *GhMIPS1D* in the transgenic *Arabidopsis* lines were examined (Supplementary Figure S5). As is reported, the T-DNA insertion *Arabidopsis* mutant lines exhibit abnormal cotyledon development (Figure 5B), shorter plants (Figure 5F) and increased inflorescence branch numbers (Figure 5G), and sensitivity to strong light stress (Supplementary Figure S6). The transgenic *mips1/GhMIPS1D* lines, obtained by transforming *35S::GhMIPS1D-GFP* vector into *Arabidopsis mips1* mutants, indicated stable expression levels of *GhMIPS1D* and rescued the aberrant phenotypes of *Arabidopsis mips1* mutants (Figure 5C–G). In addition, the *Arabidopsis* mutant lines expressing *GhMIPS1D* also showed increased tolerance to light stress under 220 μmol·m^−2^s^−1^ (Supplementary Figure S6). These data suggest that *GhMIPS1D* is the predominantly expressed *MIPS* gene in *G. hirsutum* and is an active gene effectively to rescue the abnormal defects of growth and development in *Arabidopsis mips1* mutants.

### 2.4. Ectopic Expression of GhMIPS1D Promotes Root Cell Elongation in Arabidopsis

**Figure 6:**
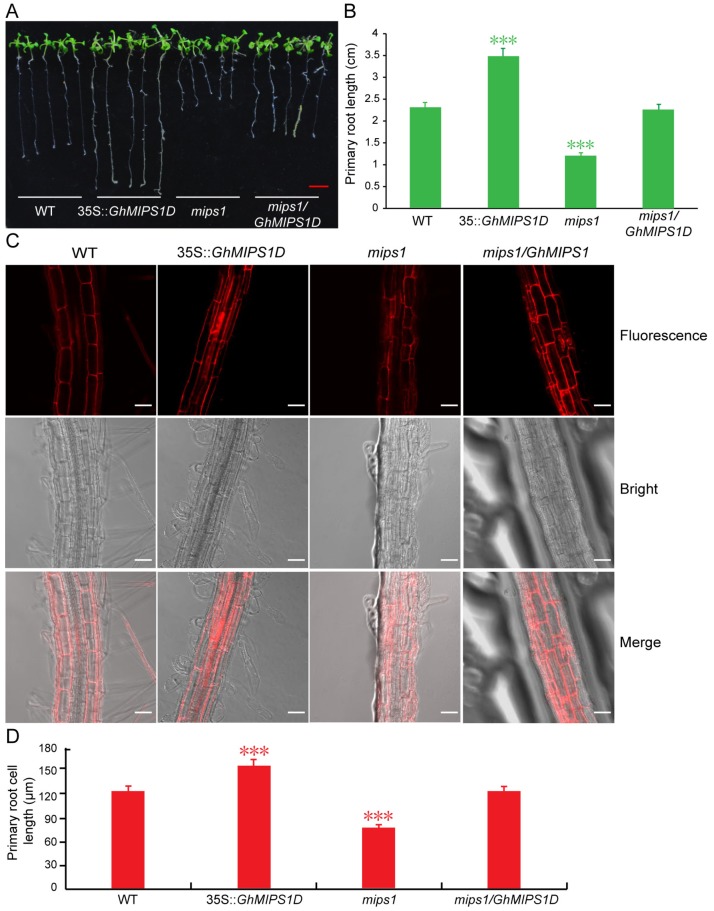
Ectopic expression of *GhMIPS1D* promotes *Arabidopsis* root cell elongation. (**A**) The primary root phenotypes of wild-type (WT), transgenic lines ectopically expressing *GhMIPS1D* (*35S::GhMIPS1D*), *mips1* mutants (*mips1*) and transgenic lines expressing *GhMIPS1D* in *mips1* mutants (*mips1/35S::GhMIPS1D*). Red bar = 0.5 cm. (**B**) The primary root lengths are measured (*n* = 10). (**C**) Fluorescence images of *Arabidopsis* root cells under confocal microscopy. Bars = 50 μm. (**D**) The root cell lengths are measured (*n* = 50). The results show that ectopic expression of *GhMIPS1D* significantly induces the cell length in *Arabidopsis* roots. ***, *p* < 0.001 compared to WT.

Considering that the Ins derivates play essential roles in root development and root hair formation [29,30], and *GhMIPS1D* gene highly expressed during the stages of fiber initiation and early elongation (Supplementary Figure S2), which indicates its potential important function for plant cell elongation. Thus, we further measured the length of primary roots and root cells in *Arabidopsis* lines ectopically expressing *GhMIPS1D*. The transgenic *mips1/GhMIPS1D* plant lines exhibited normal root length similar to that in WT, suggesting that *GhMIPS1D* is a functional gene to compensate the root elongation development defect of *Arabidopsis mips1* mutants (Figure 6A,B). In addition, the transgenic plants ectopically expressing *GhMIPS1D* in WT *Arabidopsis* demonstrated significantly increased primary root length (Figure 6A,B). Further confocal microscopy detection displayed that the primary root cell lengths are increased from 124.02 ± 10.01 μm (WT) to 152.14 ± 16.58 μm (*35S::GhMIPS1D*), indicating that *GhMIPS1D* is essential for plant cell elongation (Figure 6C,D). Totally, the genetically functional complementary analyses suggest that *GhMIPS1D* is a fully functional *MIPS* and plays as a key positive regulator to involve in cell elongation.

## 3. Discussion

Lots of *MIPS* genes have been identified in numerous living organisms, such as bacteria, fungi, animals, and higher plants, etc. [31]. Generally, there are one or two *MIPS* members in most plant species, with the exception of three members in *A. thaliana*, and of four members in *Z. mays*, *B. rapa*, *B*. *oleracea* and *G. max*, respectively. In this study, by genome-wide analysis, we identified four *G*. *hirsutum MIPS* family genes that are distributed across different chromosomes (Supplementary Figure S1). Phylogenetic tree of 70 MIPS from 36 species was constructed and used for evolutionary analysis, benefiting from the dramatically increased genome database. The protein sequences of eukaryotic MIPS exhibit high conservation, supporting the explanation that eukaryotic *MIPS* genes have a monophyletic origin (Figure 2). However, the single-cell algae *C. merolae* and *Ostreococcus tauri* possess MIPS proteins of 533 and 535 amino acids, while all higher plants have MIPS proteins of around 510 amino acids (from 509 to 511), displaying that sequence lost events had occurred after plant landing. Only two higher plant MIPS proteins possess amino acids much longer than 510 amino acids: *S. cucullate* MIPS1 (537 amino acids) and *G. max* MIPS2 (526 amino acids) (Supplementary Table S2), indicating special evolutionary events in these two species. Many studies reported the conservation of MIPS protein sequences, but few noticed the exon-intron structure evolution patterns of *MIPS* genes [32,33]. The exon-intron structure analysis presented here suggested that the *MIPS* gene structures were highly conserved in higher plants except for *Brassicales*, suggesting that *MIPS* family genes underwent more complicated evolutionary events during the *Brassicales* plants evolution (Figure 2 and Figure 3). This may explain the fact that *Arabidopsis MIPS* family members show function diversity [17,18,31,34].

MIPS is the key biosynthetic enzyme for the formation of Ins and its derivates, including PtdIns and PtdInsP, which have been proven as a crucial regulator to control plant growth and organ development. Of the four *GhMIPS* members, *GhMIPS1D*, showing the predominantly expression during the early stages of fiber development (Supplementary Figure S2), was selected to further investigate cotton *MIPS* functions of participating in plant growth and development. After ectopic expression of *GhMIPS1D* in *Arabidopsis* loss-of-function *mips1* mutants, the aberrant phenotypes of cotyledon abnormality, shorter plants, increased inflorescence branches and sensitivity to light in the *mips1* mutants were rescued significantly (Figure 5 and Supplementary Figure S6), implying the cotton *MIPS* important roles in the involvement of plant growth and development as the pivotal enzyme to produce Ins and its derivates that are important signal molecules of the cell. Knockout of the soybean *GmMIPS1* affected the early development of the embryo and resulted in the termination of seed mature that might be caused by the reduction of IP_6_ [35]. *Arabidopsis MIPS1* is essential for seed development, and the loss of *MIPS1* leads to the reduction of AsA and PtdIns and thus produces the irregular phenotypes of smaller plants, curly leaves and generation of lesions [18]. Ins produced by MIPS is crucial for *Arabidopsis* embryogenesis through regulating the synthesis of PtdIns and phosphatidylinositides and thereby involving in endomembrane structure trafficking and PIN1-mediated auxin singaling pathway [17]. The functions of the other *GhMIPS* genes should also be verified in the future for a better understanding of the diverse roles of Ins pathway in cotton. Besides, *MIPS* has also been reported to function in salt and drought tolerance, indicating that the *MIPS* gene family play various roles in plant development [36,37]. However, there still are unsolved problems should be addressed in the future, such as the mechanism leading to increased inflorescence branch numbers in *Arabidopsis misp1* mutants, as well as the possible phenotypes of loss-of-function cotton *mips1* and/or *mips2* mutants.

Due to the highly conserved protein sequences of GhMIPS1D and AtMIPS1, it is not surprising that *GhMIPS1D* could rescue the *Arabidopsis mips1* mutant phenotypes. As is well known, MIPS proteins from diverse species share highly conserved core catalytic domains, which endow the activity to catalyze rate-limiting redox reaction to generate the Ins formation from G6P [31]. The *G*. *hirsutum* MIPS possessed several typical domains that serve as binding and catalysis of MIPS proteins (Figure 2, Supplementary Figure S3). Since the first MIPS was reported from *Archaeoglobus fulgidus*, many MIPS proteins have been investigated from different organisms. The MIPS in higher organisms usually showed cytosolic or organellar location. In this study, subcellular distribution analysis indicated the location of GhMIPS1D in the nucleus, plasma membrane and endomembranes (Supplementary Figure S4), suggesting that cotton MIPS may perform a diverse role in different compartments to participate in plant growth and development. By suppressing the spreading of heterochromatin, MIPS can bind to its promoter to induce its own expression, providing the illustration of the regulatory mechanism at the transcriptional level [38]. 

MIPS catalyzed Ins biosynthesis provides the important supply for synthetizing its derivates containing PtdIns, PtdInsPs and InsPs that are vital signal molecules to affect cell growth by participating in cellular signal transduction [39]. In this study, *GhMIPS1D* indicated preferential expression during fiber initial growth stage (Supplementary Figure S2), and ectopic expression of *GhMIPS1D* in *Arabidopsis* rescued the shorter primary root lengths in *mips1* mutants and promoted the root cell elongation in WT, respectively (Figure 6). Severe reduction of IP_6_ content in *Arabidopsis ipk1-1* mutants significantly inhibits the root hair elongation [40]. The decrease of PtdInsP_2_ content in *Arabidopsis* results in suppression of pollen tube elongation by affecting apical pectin deposition and membrane trafficking [41]. Although many Ins derivates have been reported to be involved in cell growth, whereas, this is the first time to prove that *MIPS* family members could promote the root cell elongation in *Arabidopsis* (Figure 6). This phenomenon is reasonable for that previous work showed that pectin and AsA play important roles in cotton fiber elongation [25,26]. *MIPS* has been reported to increase the synthesis of pectin precursor of UDP-d-glucuronic acid (UDP-GlcA) and AsA [42]. UDP-GlcA, PtdIns and PtdInsP that are important Ins derivates have been studied to involve in fiber elongation growth as important components of the cell wall and cell membranes [25,28].

De novo synthesis of Ins is required for the correct transport and localization of auxin during embryo pattern formation in *Arabidopsis* [17,18]. The functions of *MIPS* during cotton fiber development has not been well studied before. Many studies have reported that auxin signaling is essential for the initiation and early elongation of cotton fiber [43,44]. In the present study, *GhMIPS1D* was highly expressed at the early stage of cotton fiber development (Supplementary Figure S2), providing the potential possible direct or indirect link between MIPS catalyzed synthesis of Ins and its derivates and auxin signal transduction. *Arabidopsis mips1* mutants exhibit a significant reduction of PtdIns content and decrease of auxin polar transport rate that regulates the cellular auxin distribution [45]. Further investigation on *MIPS* and phytohormone crosstalk will provide a better understanding of functions of *MIPS* during cotton fiber development in the future.

In conclusion, we identified four *MIPS* family gene members in *G. hirsutum*, the phylogenic relationship, multiple sequence alignment, and motif distribution analyses showed that the *MIPS* gene family is highly conserved. Further genetically functional complementary analysis showed that *GhMIPS1D* is the predominantly expressed *MIPS* gene in cotton fibers, and ectopic expression of *GhMIPS1D* in *Arabidopsis mips1* mutant rescues the phenotypes of abnormal cotyledon development, increased inflorescence branch numbers and light stress sensitivity. Moreover, ectopic expression of *GhMIPS1D* in WT *Arabidopsis* promotes root cell elongation significantly. Our results provide a genome-wide analysis of the *MIPS* gene family in *G. hirsutum* and suggest that the *GhMIPS1D* is a positive regulator involving in plant cell elongation.

## 4. Materials and Methods

### 4.1. Sequence Acquirement and Chromosomal Distribution of GhMIPS Genes

The prokaryotic MIPS protein sequences were retrieved from NCBI (database downloaded on 31 December 2018) by online BLASTP. The eukaryotic MIPS protein sequences were determined using local BLASTP program by submitting *Arabidopsis* MIPS to the genome database for each organism. The genome data were downloaded from the online database Phytozome (v12.1.6). The candidate MIPS proteins were confirmed by screening the conserved domains using InterProScan. The genomic location of *GhMIPS* genes was performed using local BLASTN against the *G. hirsutum* genome database. Mapinspect was used to draw the location of *MIPS* genes in different chromosomes.

### 4.2. Phylogenic and Evolutionary Analyses

Multiple sequence alignment was performed using ClustalW and the phylogenetic tree was constructed by MEGA5.0 [46] using the neighbor-joining method with bootstrap tests 1000. The evolutionary analyses were performed as previously described [47,48]. Briefly, the exon-intron distribution analysis was performed using the gene structure display server (GSDS, http://gsds.cbi.pku.edu.cn/index.php). Conserved motifs were recognized by MEME online software (http://meme-suite.org/tools/meme). The *Ka/Ks* was calculated by Dnasp (v6) software [49].

### 4.3. Plant Materials

The *Arabidopsis mips1* mutant (SALK_023626) was obtained from Lijia Qu, State Key Laboratory of Protein and Plant Gene Research, College of Life Sciences, Peking University. All *Arabidopsis* plants were grown on 0.5 × MS medium as previously described [17].

### 4.4. Construction of Vectors, Subcellular Localization Analysis and Ectopic Expression of GhMIPS1D in Arabidopsis

The full-length of the coding sequences of *GhMIPS1D* gene was amplified using primers in Supplementary Table S3, and then cloned into the modified pCAMBIA2300-GFP vector using *Kpn*I and *Xba*I to generate *35S::GhMIPS1D-GFP*, which was used for further ectopic expression and subcellular localization analyses. The successfully constructed vectors were then transformed into *Agrobacterium tumefaciens* stain GV3101.

The subcellular localization analysis was performed using onion epidermal cells and *A. tumefaciens* containing *35S::GhMIPS1D-GFP* vector. Briefly, the inside epidermal layer of the onion was separated. After soaking in 75% ethanol for 10 min and washing 3–4 times with sterile water, the inside epidermis was cut and co-cultivated with *A. tumefaciens* containing *35S::GhMIPS1D-GFP* vector on 1/2 MS at 28 °C under darkness. After a sub-culturing for 24 h, a confocal laser-scanning microscope (Zeiss LSM510, Oberkochen, Germany) was used to detect the GFP signals with an activation wavelength of 488 nm.

Wild-type (WT) and *mips1* mutant *Arabidopsis* plants were transformed using *A. tumefaciens* stain GV3101 containing *35S::GhMIPS1D-GFP* vector. The functional analysis of transgenic *Arabidopsis* was performed as reported before [50].

### 4.5. Statistical Analysis

All statistics were performed by One-way ANOVA followed by Bonferroni test using SigmaStat software (Version 4.0) (Starcom Information Technology Ltd, Bangalore, India). *** represents significant difference at *p* < 0.001 level.

## Supplementary Materials

Supplementary materials can be found at https://www.mdpi.com/1422-0067/20/5/1224/s1.

## Abbreviations

APX	Ascorbate peroxidase
AsA	Ascorbic acid
BLAST	Basic local alignment search tool
DPA	Day post anthesis
G6P	d-glucose 6-phosphate
GlcA	d-glucuronic acid
IMP	*Myo*-inositol monophosphatase
Ins	*Myo*-inositol
IP	*Myo*-inositol polyphosphate
MEGA	Molecular evolutionary genetics analysis
MIPS	*Myo*-inositol-1-phosphate synthase
MS	Murashige and Skoog
PtdIns	phospholipid phosphatidylinositol
PtdInsP	phosphoinositide phosphate
RNAi	RNA interference
UDP-GlcA	UDP-d-glucuronic acid
WT	Wild-type
