# Supplementary material for: A Cotton (Gossypium hirsutum) Myo-Inositol-1-Phosphate Synthase (GhMIPS1D) Gene Promotes Root Cell Elongation in Arabidopsis
Source: Int J Mol Sci. 2019 Mar 11;20(5):1224. doi: 10.3390/ijms20051224 (PMC6429088; doi:10.3390/ijms20051224)
Supplement: Supplementary file 1 [file ijms-20-01224-s001.zip › Supplementary files_final/Supplementary Figures-R2_final.doc]

**Supplementary Materials**

**Figure S1.** **Chromosomal distribution of *G. hirsutum* *MIPS* genes.** The four *GhMIPS* genes mapped to the chromosomes of A sub-genome and D sub-genome, respectively. Chromosomes were represented by different scales. Red lines indicate the duplication gene pairs.

**Figure S2. The expression level of GhMIPS transcripts determined by RNA-seq data.** The FPKM values were calculated from RNA-seq data of cotton ovules (0 day post anthesis, 0 DPA), fibers (3 DPA, 10 DPA and 15 DPA), and leaves. RNA-seq data were downloaded from NCBI Sequence Read Archive (SRA) under accession SRA180756 and Fragments per Kilobase of exon model per Million mapped reads (FPKM) were calculated using Cufflinks (Version 2.1.1) [1].

**Figure S3.** **Detailed amino acid sequences of ten conserved motifs of MIPS.** The height of each letter represents the frequency of amino acid at that position, and the length indicates the number of amino acids in each conserved motif.

Supplementary Figure 4

**Figure S4.** **Subcellular location analysis of GhMIPS1D.** *GhMIPS1D::GFP* was transformed into onion epidermal cells using the *Agrobacterium*-mediated method. Mannitol was used to induce plasmolysis. Images presented under bright, fluorescence, and merge conditions are indicated by confocal laser-scanning microscopy. Bars = 50 µm.

**Figure S5.** **RT-PCR detection of *GhMIPS1D* in transgenic and wild-type *Arabidopsis*.** Materials of transgenic lines and WT were used for total RNA extraction and subsequent RT-PCR analysis. *AtActin* was used as reference gene.

**Figure S6.** ***GhMIPS1D* rescues the light stress sensitivity of *Arabidopsis* *mips1* mutant.** *Arabidopsis* WT, *mips1/GhMIPS1D* and *mips1* plants grew under different light intensity conditions. Leaves of *mips1* mutants appeared lesion-mimic patches under the high lights compared with WT and *mips1/GhMIPS1D* transgenic plants. Bars = 1 cm.

**Reference**

1. Li, F.; Fan, G.; Xiao, G.; Zou, C.; Kohel, R.J.; Ma, Z.; Shang, H.; Ma, X.; Wu, J.; Liang, X.; et al. Genome sequence of cultivated upland cotton (*Gossypium hirsutum* TM-1) provides insights into genome evolution. *Nat. Biotechnol.* **2015**, 33, 524–530.
